# Carving out space for collective action: a study on how girls respond to everyday stressors within leisure participation

**DOI:** 10.1080/17482631.2020.1815486

**Published:** 2020-09-20

**Authors:** Anne Christina Gotfredsen, Isabel Goicolea, Evelina Landstedt

**Affiliations:** a Department of Epidemiology and Global Health, Umeå University, Umeå, Sweden; b Umeå Centre for Gender Studies, Umeå University, Umeå, Sweden; c Department of Social and Psychological Studies, Karlstad University, Karlstad, Sweden

**Keywords:** Youth mental health, leisure participation, case study, stress process, social practice theory, participatory observations, photo elicitation, thematic analysis

## Abstract

**Purpose:** Stress and achievement pressure constitute factors affecting young people’s mental health, especially among girls. Leisure participation holds the potential to be a collective space where young people can respond to stressors together. This study explores how girls collectively construct responses to daily stressors within the context of leisure participation.

**Methods:** Nine focus groups were conducted with 16 girls aged 14–21 who were active members in two sport organizations in northern Sweden. Data was collected by using participatory observations and photo-elicited focus group discussions.

**Results:** Our findings from the inductive thematic analysis were interpreted by combining the stress process model with social practice theory, resulting in three subthemes or responses: sharing sites of responsibility, resisting norms related to (gendered) youth and focused distraction. The subthemes were abstracted into the central theme of trustful belonging as a resource for collective responses, representing what pre-conditions need to be in place to make the responses possible.

**Conclusion:** Leisure participation is an important relational space for young people to respond to stressors by making use of everyday routines, and the agency these social practices hold. However, the effort needed to respond to these stressors brought additional pressure in terms of responsibilities, and achievements.

## Introduction

The reasons why young people increasingly suffer from mental health problems such as anxiety and depression are widely debated today, as is the best way forward to turn this development around (Burger & Samuel, 2017; Patton et al., 2016). The complexity of social, environmental and relational factors such as gender and sexuality, socio-economic status, peer and family relationships, exposure to harassment, social media, and the broader social structures of education and employment patterns are important aspects shaping the mental health of youth (Landstedt & Coffey, 2017). In Sweden, stress and stress-related mental health complaints are common among young people and have been increasing over the past few decades (Anniko et al., 2019). Scholars tend to agree on the importance of looking beyond the level of the individual towards the socio-structural context when exploring trends and differences in these health outcomes (Bowleg, 2012). For example, drawing on the stress process model (Pearlin et al., 1981), young people’s stressors are located within their broader social context where structural positions such as racialization, social class, and gender shape exposure and responses to stressors (Mcleod, 2012). Numerous studies have shown that achievement pressure affects young people’s stress levels, and that here the educational system is one of the main stressors, especially among girls (Persike & Seiffge-Krenke, 2012; Wilhsson et al., 2017). Girls and young women face multiple stressors related to pressure on performance and educational success, caring for and adapting to the needs of others, and a constant struggle for social acceptance, visibility, and value in relation to their body, appearance, and achievements (Anniko et al., 2019; Strömbäck et al., 2014). These demands need to be understood in relation to socio-structural constructions of gender in a neoliberal context, which emphasizes the responsibility of the individual and makes demands on young people to be independent, self-inventive, flexible, and responsive in order to be successful (Budgeon, 2011; Landstedt & Coffey, 2017; West, 2017).

In a study on gender-sensitive stress management interventions, Strömbäck et al. (2013) highlighted the need to reduce the individualization of health problems among girls and instead encourage spaces for collective support, action, and change (Strömbäck et al., 2013). This follows the stress process model, which, in addition to stressors, also identifies the resources to which young people have access, especially highlighting social support as a buffer against stressors (Mcleod, 2012). A sense of belonging and coherence, with social support from similar others within a social context have been identified by scholars as positive mechanisms for physical and psychological health (Baumeister & Leary, 1995; Moksnes & Espnes, 2014; Thoits, 2011; Wilhsson et al., 2017). We argue that leisure participation among young people has the potential to be such a context or space for social support, where young people can respond collectively to the stressors experienced in their daily lives. The aim of this study is therefore to analyse how girls collectively construct responses to everyday stressors within the context of leisure participation, using a multiple case study based in northern Sweden.

Leisure participation, social practice theory, and young people’s agency

Positive leisure activities are a core ingredient of overall wellbeing (Kuykendall et al., 2015; Lee et al., 2012; Newman et al., 2014). In a study by Haraldsson et al. (2010) on what makes life less stressful for adolescent girls, leisure was perceived by the participants as a basis for recovery and enjoyment. Civic engagement (such as political participation) and leisure have also been found to be important sources of belonging, which is positive for young people’s mental health and social development (Bruner et al., 2017; Landstedt et al., 2016). Although this specific study situates young people’s leisure within the context of organized leisure participation such as sport and physical activity, we understand leisure participation as a broader and more encompassing concept where the division between organized and unstructured leisure is not always clear-cut. As Spracklen et al. (2017, p. 10) write, “leisure is games, stories, discussions, eating, drinking, moving, painting, playing, making music, reading and watching things.”

However, research has also pointed out inequities in relation to leisure participation, whereby gendered expectations and experiences of stress and wellbeing influence young people’s participation (or non-participation). Inequalities based on dis/ability, gender identity and expression, ethnicity, sexuality, geographical location, and socio-economic background come into play in terms of having access to safe and positive leisure activities (Ferris et al., 2013; Lindström & Öqvist, 2013; Swedish Agency for Youth and Civil Society [MUCF], 2014). As a final note, the relationship between young people’s mental health and leisure participation does not always have a positive character. Although studies on educational stress and leisure are limited, research indicates that participating in competitive and performance-focused activities (e.g., sports, music) can create negative experiences for young people in terms of increased stress, achievement pressure, and anxiety (Merkel, 2013; Wilson et al., 2010).

Yet, the ways in which leisure participation can potentially counter the negative consequences linked to stress and achievement pressure deserve further investigation, especially among girls. More specifically, we would like to highlight the potential resources relating to leisure participation in terms of social support and the possibility for change and agency in relation to the stressors that girls are facing. We connect these resources to social practices containing elements of agency and change (Schatzki, 2002). Social practice theory defines social practice as a routinized behaviour involving interconnected elements of bodily and mental activities, together with shared competencies, knowledge, and skills (Caldwell, 2012; Maller, 2015). Practices are nevertheless shaped by the wider realm of power relations and society, while each practice also acts to shape these wider aspects of social systems (Giddens, 1984). Crawford (2006) connects this to how individual health practices and the individual responsibility for health have become a model of and for the restructuring of neoliberal society. In line with this discussion, the concept of young people’s agency has been criticized by youth researchers for usually referring to active subjectivity, “free will”, and a celebrated, predetermined resistance to structural constraints, portraying young people who do not resist as lacking subjectivity (Coffey & Farrugia, 2014). In this study, we therefore use Kennelly’s understanding of agency as *relational* (Kennelly, 2009), directing attention towards the interactive and collaborative development of young people’s agency, between different individuals rather than within a single individual (Roholt VeLure & Baizerman, 2019).

## Methodology

We adopted an ethnographic, multiple case-study approach (Yin, 2014) to create a deeper understanding of how the participants collectively constructed responses to everyday stressors, and how the surrounding contexts interact with these social processes. The cases in this study were two groups of girls and young women (age 14–21) engaged in leisure organizations in rural Northern Sweden. The complexity of the phenomenon under investigation required a methodology in which the young people’s own experiences, situations, and strategies are central, both in terms of the data collected and the knowledge production itself (Leonard & McKnight, 2015). To capture this complexity, data were comprehensivley collected through participatory observations in addition to focus groups using photo elicitation.

We decided to use focus groups to emphasize how young people respond to the everyday stressors they face together. This method allows us to explore how social processes and different topics are elaborated upon, a process which has been termed *collective sense-making* (Braun & Clarke, 2013). Our decision to include photo elicitation was mainly in line with what Leonard and McKnight (2015) emphasize as the possibility to pave the way for wider dialogue on aspects of social life and to reduce some of the power dynamics between adult researchers and young participants. Participatory observations provided the opportunity to focus not solely on the social processes between the participants but also on the interaction between the contexts of our cases and these social processes (DeWalt & DeWalt, 2011; Guest et al., 2013).

## Study setting and case selection

The study was conducted in Northern Sweden,^1^ which covers more than half the country, but comprises only about 12% of the total population, and where 47 out of 54 municipalities are considered rural (Statistics Sweden, 2017). A number of organizations providing leisure activities for young people in rural municipalities were contacted by email with information about the project. Two sports organizations—one non-competitive and individual-based, and the other a competitive team-based sports club—expressed interest in participating and were chosen for the study. When contact was established, a more extensive project description was provided and initial meetings with the chairpersons of the two organizations were set up. Together with these key persons, further information meetings were arranged with the young members of the organizations, their parents, and other adults (e.g., coaches). Information about the study´s ethical approval and how to get in touch with the research group was distributed by email and members who were interested in participating in the study contacted the first author (ACG), who was responsible for data collection. The organizations and cases were not selected on the basis of any mental health interventions, nor previous experiences of stress and achievement pressure. The two cases, and the data collected in each case, are presented in Table I.

**Table I.** Participants, sources of data, and methods of data collection.

Case	Short description	Data collected
Team Horses (TH) Five participants Non-competitive and individual based sports club	A close group of five girls aged 14–17 years from an equestrian club, located in a small rural town. The group has known each other for many years and are engaged in multiple activities at the club, such as daily care of the horses, organizing riding classes for younger children, fundraising and taking riding lessons themselves. They also spend a lot of time hanging out at the club, doing social activities together not related to horses. Four of the participants go to the same local secondary school. The context of the case is the equestrian club, which for many years has offered riding lessons for children, teens, and adults, in addition to boarding privately owned horses.	Transcripts from three focus groups (with four or five of the participants present during each interview) using photo elicitation, in addition to approx. 50 hours of participatory observations on location. In total 194 minutes recorded interviews, generating 102 pages of transcribed material.
Team Ballgame (TB) Eleven participants Competitive and team-based sports club	A group of eleven girls and young women aged 14 to 21, who are all part of the same sports team in a small rural town. The team, which is one of the best teams within the club, has played together for about two years. The team practices three times a week, in addition to playing in competitions every other week, sometimes at home but equally often in different places around Sweden, which requires a lot of travelling. They have their weekly practices at the local sports hall, often quite late since many of them commute back and forth to school. About half of the team go to the same local school but the rest attend upper secondary school at another location. The team members hang out before practice and competitions, in addition to the many hours of travelling. The club has a strong connection with the local community and their weekend competitions and activities often attract a crowd.	Transcripts from six focus groups (the team was divided into three groups, with each group conducting two rounds of interviews) using photo elicitation, in addition to approx. 60 hours of participatory observations on location, but also at other places when travelling. In total 201 minutes recorded interviews, generating 113 pages of transcribed material.

## Collecting data

All data were collected between October 2017 and December 2018 by the first author (ACG), a PhD student who identifies as a woman from Southern urban Sweden with prior experience of conducting qualitative research with young participants, as well as engagement within the Swedish civil society. All fieldnotes and interviews were transcribed verbatim by the same author (see Table I for an outline of the data-collection process).

### Focus groups and photo elicitation

The focus groups were moderated by the first author (ACG), who distributed guiding questions to the potential participants beforehand. These questions were related to perceptions of health and stress, their engagement in the organization, social activities within the group, and collective support. To allow for a deeper level of communication between the participants, as well as with the researchers, the method of photo elicitation was added to the focus groups, encouraging the participants to answer the questions by taking photos. Photo elicitation entails the process of looking at photographs with the participants in order to evoke memories or stories, or to initiate a discussion (Mannik & McGarry, 2017). Conversations that take place around photographs can reach a more collaborative level of communication between the researcher and participants, since the participants decide which photos to choose and include in the research situation (Harper, 2002; Mannik & McGarry, 2017). Photos were sent electronically by the participants, printed by the researcher, and brought to the focus group discussion, where they served as a starting point for the conversations through open-ended questions (see Table IIIA in appendix for a more detailed description of the pictures included in the paper). The focus groups lasted between 25 and 75 minutes, were recorded, and were all conducted in Swedish at the location of the organization (see Table I).

### Participatory observations

Participatory observations were undertaken with *Team Horses* at the club and in its close surroundings, and with *Team Ballgame* at the sports hall but also while travelling to different cities in Sweden. Daily activities, feelings, and interactions between the participants were observed and reported in written and audio notes. Visual fieldnotes e.g., drawings and photos, were produced to enhance the recordings and memory while collecting data (Mannik & McGarry, 2017). One is not merely a passive observer when conducting observations, but as a researcher one assumes a variety of roles along a continuum of participation (e.g., from passive participation to moderate and complete participation) during the fieldwork (DeWalt & DeWalt, 2011). This was evident in the two cases where the opportunity and possibility to actively participate in the different activities varied, depending on contextual, ethical, and practical factors.

### Data analysis

All data from each case was considered as a separate data set and analysed according to the principles of thematic analysis (Clarke & Braun, 2017). Our analysis started while transcribing the interviews and was conducted in parallel with continued data collection, creating a dynamic development within the analytical process as we moved back and forth between the analytical and data-generating steps. To allow for different ideas and theories to be built up from the data, the ethnographic research process is necessarily inductive (Murchison, 2010), and this approach also guided our coding process. This process was conducted with help of the software program MAXQDA 12. Two separate coding systems were developed for each data set. From each of these coding sets, provisional themes, or so-called *candidate themes* (Braun & Clarke, 2013), were constructed. These candidate themes were then used to construct a model from which the “thick case descriptions” (Ellingson, 2013) were written for each case. “Thick description” is a term used to characterize the process of paying attention to contextual detail in observing and interpreting social meaning when conducting qualitative research, which also establish trustworthiness of the study (Mills et al., 2010). With the help of these thick descriptions, we continued the analysis by defining and refining new and previously identified themes in both of the descriptions, capturing both similarities and differences between the cases (see Table IIA in Appendix for an illustration of the analytical process).

### Ethical considerations

This study was approved by the Regional Ethics Committee in Umeå (2016 466–31). In keeping with the ethical standards of the 1964 Helsinki Declaration and its later amendments, the participants received information both verbally and in written form about the study’s purpose, our research procedure, and the confidentiality of the collected data. All potential participants were informed that participation was voluntary and that they could decide to withdraw at any time, without giving any explanation. In addition to the information letter previously distributed to the participants, informed consent was discussed and orally collected. Written parental consent was collected for participants under the age of 15. Prior to data collection, the participants met with the first author to discuss the methodological and ethical steps of the study, especially in relation to photo elicitation and participatory observations. The ethical guidelines set out by Cox et al. (2014) on visual research methods were used to guide the session on photo elicitation, giving special attention to consent and confidentiality. The participants could choose whether to share their pictures or not to the rest of the group, and were reminded of this option before each interview. In relation to the participatory observations, focus was directed towards the question of transparency, as to when and how observations would be conducted, what the focus would be, and note-taking, in line with Madden (2010) suggestions. Above all, the right to privacy for the participants (DeWalt & DeWalt, 2011) was stressed so they felt comfortable about saying if and when they did not want the researcher to be around. In *Team Ballgame*, this was also further discussed with the coaches. Efforts were made to secure confidentiality in terms of which photos to include in the article and by replacing the names of individuals and organizations with pseudonyms.

### Findings

**Figure 1.**
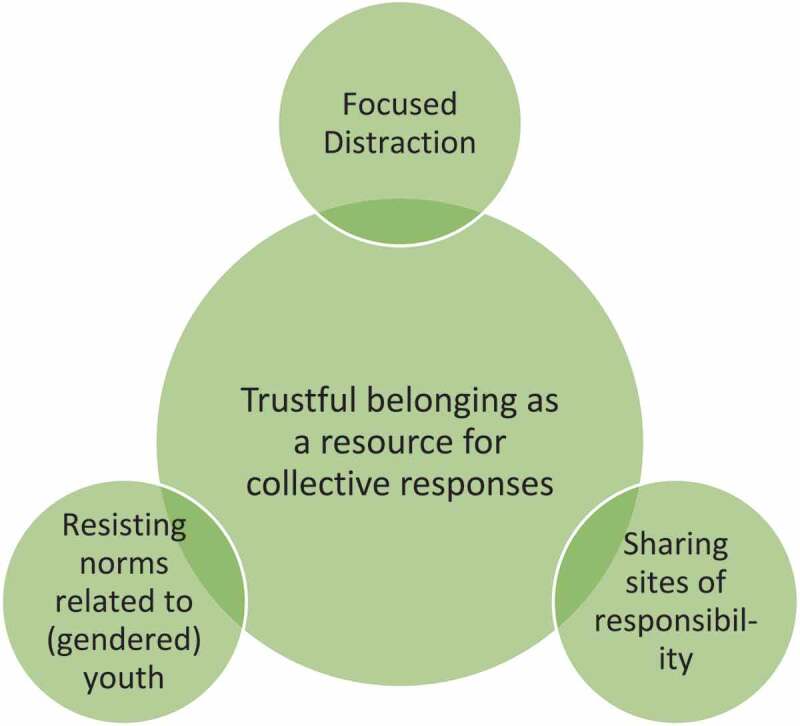
Illustration of the constructed themes.

The analysis of the fieldnotes and transcripts resulted in one central theme and three subthemes related to the collective responses constructed by the participants to the everyday stressors they are facing (see Figure 1). The three subthemes: *sharing sites of responsibility, resisting norms related to (gendered) youth* and *focused distraction* can be seen as the collective responses constructed by the participants to the everyday stressors they face. The central theme of *trustful belonging as a resource for collective responses* illustrates how these responses are created and what pre-conditions or requirements need to be in place to make the responses possible.

Girls’ collective responses to everyday stressors

### Sharing sites of responsibility

**Figure 2.**
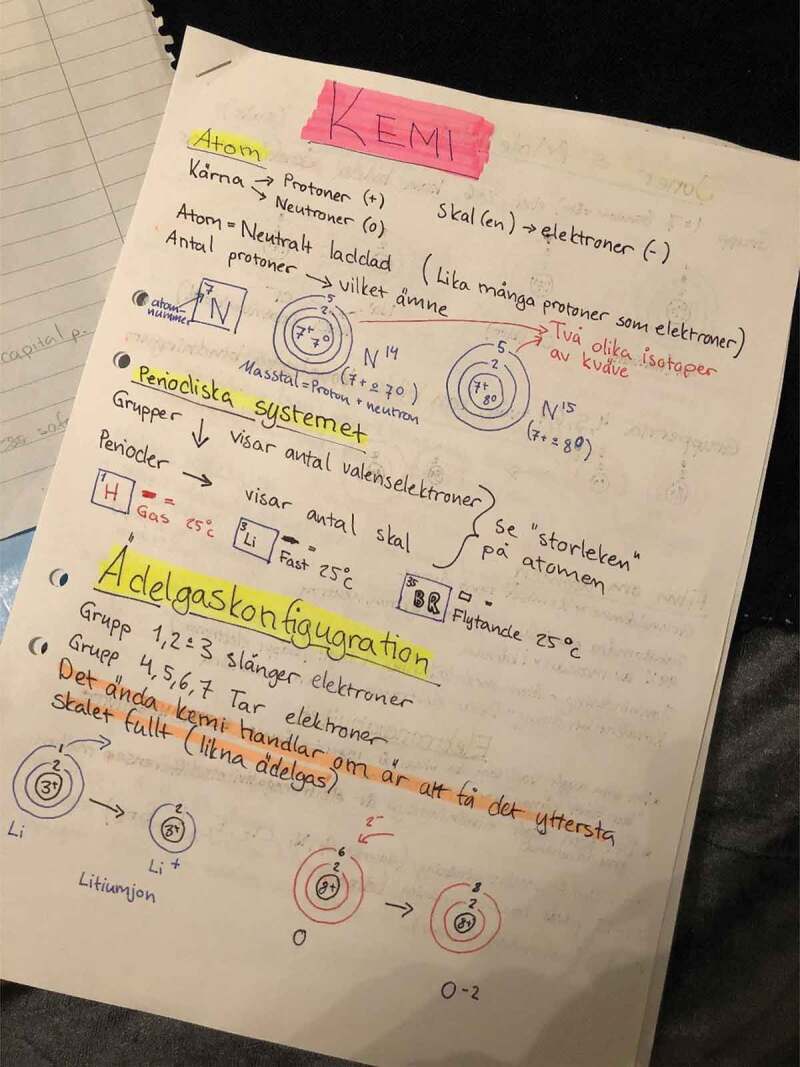
Educational pressure.

School and educational pressures were identified by participants in both cases as an important site of responsibility and as major sources of stress. In answer to the question: “what influences your health and wellbeing in a negative way?” Lisa from Team Ballgame (TB) shared a picture of her latest homework (Figure 2). She described her exhaustion in relation to school performance: “You’re so tense all the time and it just kills your brain and body!”

Educational failures resulted in feelings of disappointment, frustration, self-blame, and worry about the narrow margins in relation to future prospects, as stressed by Linnea from Team Horses (TH): “If you get a single F, you’re screwed.” Leisure participation was also identified as an additional site of responsibility: “Just the daily stress of not having enough time to see your family, or your friends … and then the homework which is always at the back of your mind … and then you have to perform in the game as well” (Veronika, TB). Being both a successful student and a successful player were important goals for Team Ballgame, meaning that the girls struggled to find enough time for both studies and practice. “It’s this constant pressure to perform … and be successful” (Lisa, TB). School was also an important site of responsibility for the girls in Team Horses, but the pressure they experienced in relation to their leisure was more related to keeping the stables and its organization up and running, rather than competing or being a successful rider.

By sharing personal experiences of these daily stressors with each other, participants turned individual struggles and worries into collectively shared experiences. When the girls got together at the stables or at the sports centre, there was room for joint reflection, debriefing and shared experiences in relation to school achievements and upcoming tests. In both teams, the girls helped each other with assignments, or comforted each other through jokes and encouragement. When Lisa (TB) had to leave early one evening to prepare for an exam, Josefine said seriously: “Remember Lisa, you don’t always have to be number one.” Although the participants clearly expressed the difficulties of reducing the demands and expectations they placed on themselves, the girls shared these experiences with each other and often talked about these challenges, as explained by Ellen (TB): “You know you’re not alone, we all struggle with the same things.”

### Resisting norms related to (gendered) youth

Through their leisure participation, the girls in both cases created resistance to social norms surrounding adolescence. Team Horses often returned to the issue of substance abuse, and how they saw young people in their community ending up in what was described as “bad company”. Linnea and Wera discussed this:

Wera: There aren’t so many people here in town to hang out with … you have to find a group or be by yourself.

Linnea: Yes, it’s very clustered … some kids are the “cool ones” (making air quotes with her fingers) and others are more … odd.

[…]

Wera: More “cool” (illustrating air quotes with her fingers) … smoking and binge drinking.

Linnea: Yeah, and then you want to fit in … I guess.

Moderator: (to the group) Do you ever feel pressured to drink or smoke?

(pause)

Wera: Not us … we have each other! We’re crazy enough as it is, we don’t need alcohol (laughs).

In terms of pressure to drink alcohol or use other substances, the participants in both cases expressed strong opinions regarding how these norms affected others in their community, but not so much themselves. As exemplified in the previous quotation, being part of a group and support system helped these girls to resist this kind of pressure. The girls in Team Horses connected this to their friendship, while in Team Ballgame it was more about having a responsibility towards the team and the club’s image not to drink, but also, as Jennifer simply put it: “You just don’t have time to drink when you’re an athlete!”

Norms related to gender and femininity were also challenged and partly resisted in the two cases. In particular, the girls in Team Horses invested a lot of time in keeping up with social media and managing their online personas, a topic they often discussed when hanging out at the club. They described how this interaction brought joy and inspiration, but also how the constant exposure to certain beauty and body norms made them feel, something the girls in Team Ballgame also discussed.

Moderator: Do you think these kinds of norms regarding beauty and body ideals affect you?

Judith: Yes!

Moderator: Okay … in what way?

Judith: Or … you think about what you eat … sometimes … or perhaps it’s just me? [to the others in the group] Yes, perhaps it’s just me …

(pause)

Ellen: No, I do it too … a little bit.

For Team Horses, being part of the group and the environment at the club was recognized as creating distance from the social demands of how to look and behave. The girls shared how they were sometimes mocked at school for being horse girls, and by this definition not being feminine enough. The stables were a social space where one could relax and let “your hair down” and not worry about looks. Ella said: “We spend all our time in the stables, we really don’t have time to think about that stuff [looks and appearance]!” They would joke about how long it had been since they last showered, or how muddy and sweaty they had got on their last ride. This was seen as a sign of dedication, of not being afraid of hard physical work and not being perceived as “girly”. In comparison, Team Ballgame complied with more traditional norms of femininity, such as wearing make-up during practice and fixing their hair before and after a game. However, there was also room for skipping showers and joking around with each other about being sweaty, and having a messy look, especially among the more senior players.

### Focused distraction

**Figure 3.**
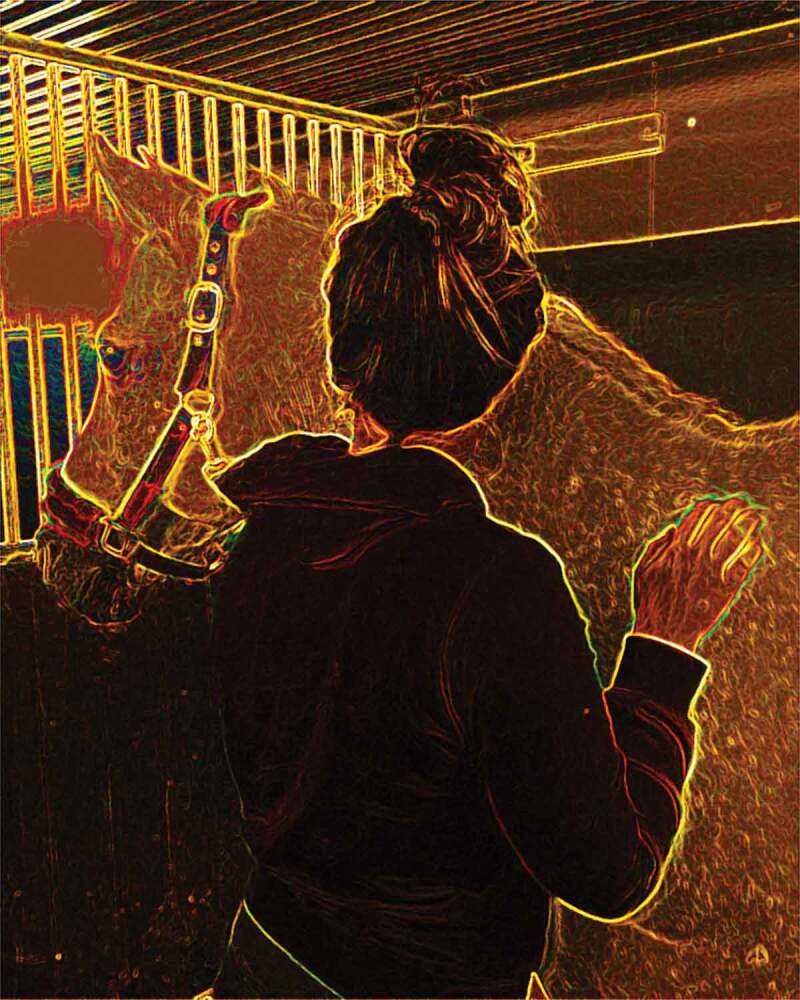
Focused distraction.

This theme represents a response in which participants were distracted from everyday stressors by directing their attention towards something else. By focusing on their leisure activities, the girls in both cases enjoyed a break from stress, anxiety, and worries. Team Ballgame emphasized the importance of coming together at practice and just “beasting it out” (to give all you got) while playing. As Jennifer explained: “I have a rather problematic relationship with my dad, we fight a lot … when I come here and play with the team, I just let go of everything else … it’s very, very relieving.” Practicing with the team was also illustrated as “emptying your head” and “getting some anger out of the system”. The girls from Team Horses described how their interactions with the horses, caring, cuddling, and riding, were crucial for being able to relax and create a distance from everyday stressors, for example, related to school. This was described by Linnea when discussing one of the photos taken by the participants (Figure 3):

Linnea: Around horses you have to stay focused and then you just feel less stressed … since I very easily get super stressed about minor things … for example, in school, I always want to get a full score on my tests, and then coming here to the stables makes me feel less stressed.

Moderator: So, what happens when you come here?

Linnea: I just feel, well … calmer … I can hang out with my friends and then I don’t think about the exams, and all the things that could go wrong … If I sit at home, I always start to think about all the things I should be doing and I feel guilty for not studying, but at the stables I have other things to think about … I can always help out with the horses or other things.

Moderator: That feeling, does it stay with you when you leave the stables? When you get back home?

Linnea: Well, then I start to worry again and feel stressed about the exams and assignments … but if I stay here for a very long time, then … (laughs)

In both cases, opinions differed about whether this relaxing feeling from their focused distraction stayed with them or not. Some said that the stress and anxiety returned the moment they got back home and “opened up” to troubling thoughts and worries. Others said that the feeling stayed with them for some time and that they felt happier and more relaxed, making it easier to focus on homework in the evenings. Molly (TB) shared her thoughts:

Well, of course, the many hours of practice can be an extra stressor in life, but on the other hand, it’s a moment when you can just get away … from homework or whatever and do something else for a while … and think about something else … which is also a good thing.

However, this feeling was very closely connected to achievements for Team Ballgame. If the team had had a bad day and had not played well, or if someone was not happy with their individual performance, the participants did not experience the relaxing feeling of being distracted.

Being distracted by their leisure participation was mostly perceived as an individual experience or emotion. At the same time, this response to stress was collectively constructed because the girls needed each other to create or reinforce this feeling, although it was manifested differently in the two cases. While riding or caring for the horses at the stables, the atmosphere between the girls was often calm, focused, and silent, only occasionally interrupted by someone asking for advice, or borrowing equipment from each other. The interaction with the horse was personal and individual, but at the same time, they shared these experiences with their friends around them, and this made them feel connected to the group. Team Ballgame obviously needed each other to build the team, to be able to play, and to be distracted from stressors in life, but this feeling was connected to individual achievements and did not necessarily strengthen the group as such.

The three subthemes represent the collective responses to everyday stressors as constructed by the girls in the two cases. The following section will illustrate the main theme; how trustful belonging was a necessary resource for these collective responses to be generated.

Trustful belonging as a resource for collective responses

**Figure 4.**
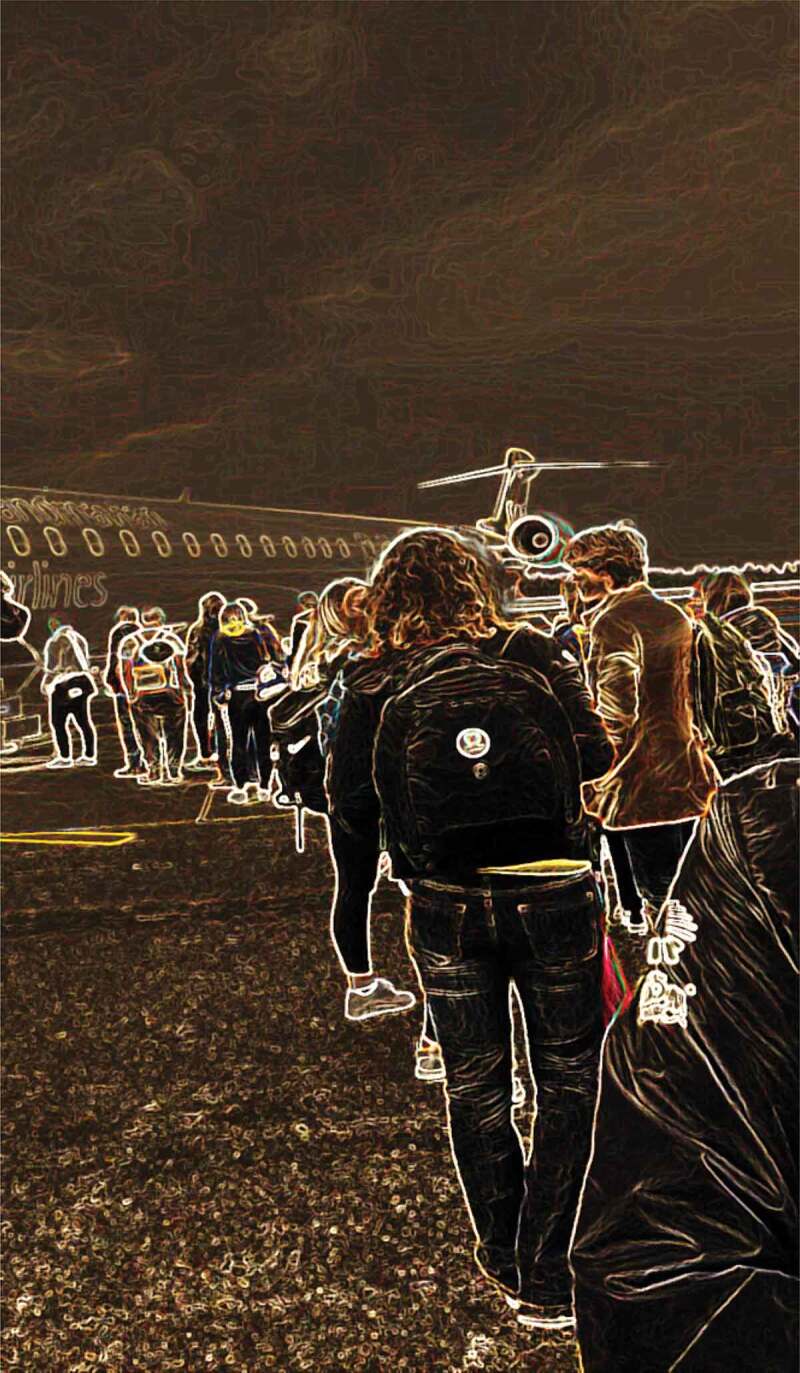
Building a sense of belonging.

When discussing the relationship between mental health and leisure participation during the interviews, both Team Ballgame and Team Horses spoke about the importance of belonging. Feeling that you belong to a certain social context, a team, and a group of people who share the same interest as you was described by Team Ballgame as positive for their wellbeing. Belonging was built through spending a lot of time together: Jennifer (TB): “the team becomes your family … a second family”. When asked what belonging signifies to the girls, Johanna took a picture of the team travelling (Figure 4). She explained: “Well, belonging to me is when we travel together … then we spend a lot of time together” (Johanna, TB).

For Team Horses, belonging was more related to the physical space of the organization. The 24/7 access to the club made them feel as though they always had a place to go after school, a place where they felt they belonged. Above all, Team Horses connected a sense of belonging with friendship. They described how their shared passion for horses made them very attached to each other. During a conversation with Linnea (TH), she thoughtfully said that she does not really have any other true friends outside the club; the girls here are her best friends. Friendship was an important resource for sharing sites of responsibility, which required that one felt safe to share experiences of stress, anxiety, and achievement pressure, but also in collectively resisting and challenging gendered norms related to youth. They had a jargon (in terms of humour, expressions, and phrases used) and topics to which they often returned when hanging out, such as school, music, social media, and things that were going on in the town or with other peers, but also personal issues and problems, as explained by Wera (TH): “We help each other a lot, supporting each other with whatever problem we have (smiles) … talking about it and stuff.”

In Team Ballgame, friendship was more instrumental and not associated with belonging to the same extent. During the interviews, the girls often returned to how they were teammates, friends, and competitors, all at the same time. The tough competition within the team resulted in the girls having to deal with both disappointment and frustration at being benched, at the same time as trying to stay happy and supportive for the team. Jennifer (TB) shared her experience:

I’ve been benched for quite some time now, and of course you think “Shit, why do they get to play instead of me?” but you can’t let it show, you just have to be supportive from the bench, you think one thing and show another … otherwise the whole team will be discouraged.

For the sake of the team spirit, one had to be friends and get along: “I mean, you don’t have to hang out as friends do, but you have to be friends during the game … if not, you’ll ruin it for everyone” (Johanna, TB). In relation to this, Lisa (TB) highlighted the importance of trust: “you need trust to build a sense of belonging, to share things with each other without being afraid that the whole school will know about it the next day.” The feeling of belonging to a group and sharing the same team identity with others with whom you have a trustful relationship was crucial for responding to everyday stressors. To enjoy the relaxing sensation of focused distraction, or “emptying your head” and “beasting it out”, as it was described by the players, they relied on a positive team spirit through friendship, or at least a trusting companionship. However, the feeling of belonging was also described by some of the girls as quite fragile since it was connected to achievements and your performance as a player: “it’s like, if you’re the only one who doesn’t get to play [during a game] … then you don’t feel like you belong to the team … honestly” (Zara, TB).

## Discussion

This multiple-case study explored how girls collectively construct responses to daily stressors within the context of leisure participation. The thematic analysis resulted in one central theme: *trustful belonging as a resource for collective responses* and three subthemes: *sharing sites of responsibility, resisting norms related to (gendered) youth* and *focused distraction*.

The participants in the study struggled in particular with pressure and stress related to educational achievements and future educational trajectories. Their accounts of the expectations and demands upon them to be successful mirrors what Harris (2004) calls the idealized subject of late modernity, whereby one is required to be resilient and self-driven towards a self-made success and fulfilment. These kinds of stressors can be understood in relation to the cultural and structural context of gender and neoliberalism, in which feminine subject positions are defined through the ideals of choice, independence, and self-responsibility (Budgeon, 2011; Pearlin et al., 1981).

Within the context of leisure participation, the girls created a sense of trustful belonging, which functioned as an important resource helping them to respond to everyday stressors. Belonging was understood and practiced differently in Team Horses and Team Ballgame. The importance of place-belongingness (Antonisch, 2010) was especially visible in Team Horses, where the participants spent much of their time together at the club and described the place as “their own (self-made) youth club”. However, belonging is not necessarily connected to a physical place. In Team Ballgame, belonging was more connected to a collective identity (Futch, 2016), arising from being players in the same team. Belonging to a group of others who shared the same team identity and interest was seen as positive for their wellbeing, a finding consistent with other studies on youth sports and belonging (Bruner et al., 2017).

In accordance with Baumeister and Leary (1995), our findings show that belonging is strongly related to social relationships, where the key features of belonging are personal contacts or interactions with other people, together with an interpersonal bond or relationship marked by stability and affective concern. In Team Horses, this was manifested in friendships based on sharing and caring for each other, in combination with the many hours they spent together at the club. In Team Ballgame, friendship was more of an instrument enabling the success of the team and was necessary to make the frequent practices and competitions enjoyable. Nevertheless, trust and trusting relations with adults and other peers was identified in both cases as an essential part in developing a sense of belonging, echoing the findings by Iwasaki and Hopper (2017) where belonging to a trustful leisure space was identified as central for young people’s emotional wellbeing. Simply being part of a social network is positive, even when the people in the network do not provide explicit emotional assistance, as long as these relationships are marked by positive concern and caring (Baumeister & Leary, 1995).

## Understanding responses as social practices of agency

Drawing on the stress process model, we recognize that the stressors experienced by the girls are connected to a broader social context, including the resources to which they have access, in terms of leisure participation and trustful belonging. Importantly, this is not a passive process of “receiving” social support. The collective responses and enabling resources are actively constructed through social practice, which is in turn manifested in relational agency (Caldwell, 2012; Kennelly, 2009; Turner, 2007). Through collaborative relational processes, know-how about stressful experiences was shared, collectively recognized, and acted upon. We argue that this relational agency is crucial in creating responses to everyday stressors. It also relates to the importance of being in a setting where social support from similar others (i.e. peers with an in-depth understanding of the many dimensions and nuances of the stressful situation) can function as a stress buffer for the girls (Thoits, 2011).

By means of the relational agency of everyday routines and practices, the participants also resisted certain pressures and stresses related to social norms of adolescence. In line with Forsberg and Tebelius’ study (Forsberg & Tebelius, 2011), the stables functioned as a safe place for Team Horses, where the girls were collectively able to articulate different gender positions in relation to looks and behaviour. Although Team Ballgame conformed to more traditional expressions of femininity, leisure participation offered the possibility of resistance to prevailing social norms, including drinking and partying. Collectively resisting social norms related to gendered youth resulted in feelings of being mature, independent, and competent, which is similar to the findings of the study conducted by Forsberg and Tebelius (2011). Focused distraction was a collective experience of just going into your own bubble knowing that you shared this experience with the others around you. This contrasts with the findings of Wilhsson et al. (2017), whose participants escaped from stress by performing activities alone, such as running in the forest.

We argue that, in their everyday lives of leisure participation, not only do the girls respond to the daily stressors they face, but this social practice of responding to stressors holds elements of agency and change. Collectively, and within the context of their leisure, the girls navigate around and challenge the stressors they face and, through these practices, they also bring change into the equation. In addition, these collective responses strengthen and reinforce the sense of belonging, making it into a reciprocal process.

## Carving out a relational space for collective responses

The girls’ leisure participation provided a social context and a physical place to meet, which functioned as the foundation for the resources and responses to be created. The social processes constructed by the girls enabled the social context of leisure to transform into a space for wellbeing. This supports the findings by Denovan and Macaskill (2017), emphasizing the importance of group-based leisure for developing social relations and feelings of belonging for managing stress. Further, these transformations turn a certain physical place and features of leisure into a so-called relational space. This is described by Massey (1993) as a place that is constructed out of particular interactions and mutual articulations of social relations, social processes, experiences, and understandings, rather than being demarcated by physical boundaries. We argue that the understanding of a relational space for collective responses that is built on social and collective support and understanding can be transferred from the context of physiotherapeutic stress-management, as in the study by Strömbäck et al. (2013), into other spaces, such as young people’s leisure participation.

However, transforming leisure participation into a relational space for collective responses is demanding. We found that the girls constantly needed to invest time, engagement, achievements, and emotions to keep the space available to them and for the space to enable these responses to happen. Leisure participation holds a complex position in relation to girls’ mental health because, while it constitutes a social space where individualized but collectively shared experiences of stress and demands can be acted upon through a relational agency, at the same time, it adds to the list of stressors. This is because girls are once again constructed as responsibility-taking subjects, responsible for their own leisure and for creating responses to stressors (Brown et al., 2013). Respectable leisure activities, such as sports, become another arena of consumption, and although they hold the potential to strengthen and empower girls, they also constitute an added site of responsibility where the ideals of the so-called Do-It-Yourself Girl can be incorporated (Heywood, 2007). As our study shows, sport was constructed as a space where girls and young women learn to take responsibility for their own lives, both in terms of successes and failures. Through the accomplishments and good lessons from these spaces, girls are supposed to develop strength, health and self-confidence and apply these to future career goals (Heywood, 2007). In our study, we saw how the participants resisted some of the norms related to gendered youth in terms of not partying or using alcohol or tobacco. Although clearly positive from a health perspective, we argue that it is an example of the discourses on individualized (heterosexual), normative femininity of success, where girls are encouraged to prioritize education, future employment, and here we include leisure and care of the body, in order to achieve respectability (Heywood, 2007; McRobbie, 2009, 2015; Strömbäck et al., 2014).

Conclusively, during the complex process of carving out a relational space for collective responses, some social norms were resisted and challenged, while others were conformed to and reproduced.

## Methodological considerations

The strength of this study lies in the multiple methods employed and prolonged engagement that allowed us to better capture the complexity of the phenomenon under study. However, we need to point out some limitations. While Team Ballgame and Team Horses offered contrasting and various perspectives, both cases were part of organizations within which questions regarding mental health, stress, and achievement pressure among young people were important to the board and leadership of the organizations. We were dependent on these adult leaders in order to access the organizations and meet and inform potential participants about our study. In terms of social position, a majority of the girls were born in Sweden, with the financial ability to participate in leisure activities, resulting in certain experiences and perspectives in relation to ethnicity, gender, and class being absent. On the other hand, this particular group of young people struggled with high levels of stress and achievement pressure in their everyday lives, making them an important group to include in research on mental health and leisure. In addition, the age of the participants varied considerably, especially in team ballgame. This age span (14–21) resulted in different experiences and perspectives being captured in the material, but might also have affected the focus groups in terms of hesitance to share certain things with younger or older peers.

Asking young people with limited time to participate in a study based on time-consuming interactive and participatory methods such as photo elicitation and participatory observations also has ethical implications (Warr et al., 2016). Judging how much we could ask of our participants in terms of time and effort were examples of *microethical* decisions or dilemmas (the kinds of ethical issues that confront researchers on a day-to-day basis) (Guillemin & Gillam, 2004), constantly present and negotiated during the research process. Further, the participants might feel hesitant about sharing certain opinions or experiences when the interviews are conducted in focus groups, regardless of whether they already know each other or are strangers (Braun & Clarke, 2013). At the same time, focus groups are suitable when the aim is to capture social processes and dynamic negotiations of meaning in specific social contexts (Wilkinson, 1998), as was the case in our study. In addition, focus groups can reduce the power and control of the researcher by share numbers, while also allowing the participants to exert greater control of the conversation (Wilkinson, 1998).

Methodological rigorousness and credibility of the case study were addressed through triangulation and thick descriptions as according to Mills et al. (2010). Since both data collection and transcription of audio material was conducted by first author only, the following analytical process was conducted and triangulated by all authors who continuously discussed codes and emerging themes to capture various perspectives and interpretations. Triangulation was also achieved using different methods, thus building a robust understanding of the cases through data collection (Mills et al., 2010). As a strategy to increase the credibility of the study, thick descriptions were included to provide contextual knowledge of the two cases, and thereby paying attention to the fine-grained details in the material (Mills et al., 2010). Further, textual material (such as quotations and photos) were included in the article to help the reader determine whether our interpretations aligned with the data.

## Conclusion and implications

Based on the accounts of the girls in this study, and the application of the stress process model with social practice theory, we have illustrated how girls collectively create responses to stressors related to educational achievements and social norms of gendered youth. Firstly, by sharing sites of responsibility, individual worries and stress turned into collective experiences in which the girls knew that they were not alone in their daily struggles. Secondly, through their leisure participation and by being part of a group of peers with similar values, the participants collectively resisted certain norms related to looks and behaviour. Thirdly, by directing their focus and attention towards their activities, the participants were distracted from the daily stressors, if only for a brief period of time. The social process of trustful belonging constituted the resource and the foundation upon which these collective responses were constructed. As seen in our study, both competitive and non-competitive leisure holds the potential to provide an important relational space for responding to stressors, making use of social practices in terms of everyday routines and tacit knowledge shared by the girls, and the agency these practices hold. However, tension is also present because new stressors are created, which require new responses. The effort needed for the girls to “carve out” space for these stressors to be responded to, and for a sense of trustful belonging to be created, also put pressure on them in terms of time, emotions, responsibilities, and achievements.

Conclusively, the current study expands our understanding of belonging by highlighting how friendship, trusting companionship, physical place and collective identity are different ways trustful belonging is created. Further, it shows how trustful belonging constitute the foundation for the different responses to happen, and finally it points to the effort required to develop and maintain trustful belonging, a process described as quite fragile at times.

In terms of implications of our findings, we argue that the Swedish civil society needs to recognize the opportunities for these organizations and spaces to be caring spaces built on trustful belongingness, but simultaneously to be considerate about the risks of putting more pressure on young people. Adequate funding and training on issues related to youth mental health and stress need to be prioritized. Finally, responding to everyday stressors within the context of leisure makes the question of equal access to leisure for young people central, calling for further exploration on youth leisure, marginalization and equity.

## Note

1. We recognize that a large area of Northern Sweden is part of Sápmi, the land of the indigenous and colonized Sámi population.

## Appendix

**Table IIA.** Example of the analytical process from one of the cases.

Extract from interviews	Candidate Themes	Thick Description	Subtheme
You know, when you have time and don’t have to hurry … when you can take your time and keep grooming … as long as you want to … Yes, when I’m having classes for the younger ones I’m always early since I have to get all the horses ready and groom them … but I find it very relaxing … You can bond with the horse just through grooming and cuddling, and that will improve your riding as well … One always has to … you can’t think about anything else when you are around the horses … you have to stay 100% focused …	Having time for grooming and cuddles Caring for horse reduce stress Bonding with horse The only focus is the horse	The integrated social life (including both humans and horses) is also important as a response to stressors. The combination of physical bonding (by caring and cuddling) with horses in combination with the strong bond between the teenagers also created feelings of “belonging” and belonging to a space where everything else can be put on hold for a while (“att inte tänka på nåt annat än hästarna”). Although “Linus” described it as being only momentary relief, perhaps these pauses (“andningshål”) still can have a lasting effect on their emotional wellbeing.	Focused distraction

**Table IIIA.** Descriptions of the pictures taken by the participants.

Figure	Description
2: Educational pressure	This picture was taken by Lisa from Team Ballgame, on the question on what influences your health and wellbeing in a negative way. She illustrated her exhaustion in relation to school performance and pressure by taking a picture of her latest homework.
3: Focused distraction	The picture was taken by Maria from Team Horses in relation to the question on what can be relaxing in the stable. She described how caring for the horses made her escape troubling thoughts and feelings of anxiety.
4: Building a sense of belonging	This picture was taken by Johanna from Team Ballgame on the question what belonging signifies to the participants. It illustrates the team travelling together by plane, going to a different city for a play-off. Johanna explained how they spend a lot of time together when travelling which she finds strengthening the feelings of belonging to the team.
